# Was Glasgow 2014 inspirational? Exploring the legacy impacts of a mega-sport event via the theorized demonstration and festival effects

**DOI:** 10.1080/17430437.2019.1571044

**Published:** 2019-02-10

**Authors:** Claire Lyne Cleland, Anne Ellaway, Julie Clark, Ade Kearns

**Affiliations:** a Centre for Public Health, Queen’s University Belfast, Belfast, UK;; b MRC/CSO Social and Public Health Sciences Unit, University of Glasgow, Glasgow, UK;; c School of Media, Culture and Society, University of the West of Scotland, Hamilton, UK;; d Urban Studies, School of Social and Political Sciences, University of Glasgow, Glasgow, UK

**Keywords:** Sport, physical activity, legacy, demonstration effect, festival effect, commonwealth games

## Abstract

The potential legacy of mega-sport events to increase physical activity and sports participation among the host community has been recognized. As part of the Glasgow Commonwealth Games 2014, a longitudinal dataset was collected, focusing on the ‘Active’ legacy domain, which aimed to help the Scottish population become active and lead healthier lifestyles. The study investigated if the event changed behaviours and attitudes towards sport and physical activity among the host community through two theorized legacy pathways: (1) demonstration; and/or (2) festival effect. Results showed that the demonstration and festival effects were relevant to the community but they were largely ineffective in changing attitudes or behaviours, suggesting that, the mechanisms were operative but not effective. It is essential that future mega-sport events implement effective promotional campaigns to engage the host city and implement initiatives alongside the event to increase physical activity and sports participation in the longer term.

## Introduction

### The growing focus on a sports participation legacy

Over the course of recent decades, the potentially beneficial legacy impacts of mega-sport(s) events have been recognized by researchers, practitioners and policy makers. One prospective and healthful legacy impact is that of increased levels of physical activity and sports participation by host populations. Such a legacy is desirable if not imperative, given that ever decreasing levels of physical activity and associated chronic health problems are a global public health concern (BHF 2015). Moreover, legacy itself has become part and parcel of all major sporting events, from the ‘managerial discourse’ (MacAloon 2008) of the sport’s governing bodies to the ‘legitimizing rhetoric’ (Whitson and Macintosh 1996) of the host cities themselves (Rogerson 2016, 499).

The intended legacies of events such as the Commonwealth Games, Olympics, and FIFA World Cup generally involve greater opportunities for sports participation and increased levels of physical activity (McCartney et al. 2010; McCartney, Hanlon, and Bond 2013). Large sporting events can instigate improvements not only to local sporting facilities but also to the local natural and built environment, infrastructure and public amenities (McCartney et al. 2010; McCartney, Hanlon, and Bond 2013). Changes that come as the result of mega-sporting events may have the potential to narrow the gap in health inequalities which have previously been linked to the determinants of ill-health (Marmot et al. 2008; McCartney et al. 2010; McCartney, Hanlon, and Bond 2013).

Despite the adoption of multiple legacy domains (including sporting, urban, social, environmental and economic change) as key areas of interest in the field of mega-sporting events (multi- or singular sports), potential resultant health and social impacts are often overshadowed by economic assessments and most frequently by the cost-benefit analysis of the economic burden for the event host city, primarily relating to stadium construction and sporting facility regeneration (Cashman 2003; Kornblatt 2006; Smith 2009; McCartney et al. 2010; McCartney, Hanlon, and Bond 2013; Alm 2012).

However, the Sydney Olympics in 2000 managed to spark research in the area of physical activity and sports participation, with the London 2012 Olympics further refocusing legacy discussions through concentrating on influencing population-level physical activity and sport participation by ‘inspiring a generation’ (HM Government 2013; Kemlo and Owe 2014). Following the London 2012 Olympics, not only did the Mayor of London establish ‘A Sporting Future for London’ but, more inclusively for the United Kingdom, the Minister for Sport set out a Ten Point Plan in a bid to take the Games legacy forward and make long-term impacts in sport and healthy living (HM Government 2013). This Ten Point Plan included: community sport, school games, physical education, disability sport, elite sport, world class facilities, major sporting events, the charity ‘Join In’, and a strategy for youth and community sport (HM Government 2013).

Lasting impacts that relate to the Ten Point Plan from the London Olympics that were documented in the headline achievements included an investment of £1 billion over a four-year period into youth and community sport and £27 million for the United Kingdom to bid and host events such as World and European Championships (HM Government 2013). This investment also included the Glasgow Commonwealth Games, 2014, which had its own legacy plan, developed partly on the back of the Legacy of the London 2012 Olympics. Glasgow offered another opportunity, to go beyond what has been described as the ‘limited success in producing a legacy of increased [sports] participation’ following London 2012 (Lovett and Bloyce 2017, 9).

### Legacy pathways

The intervention effect of mega-sport events has been theorized to occur, or rather to be activated, via two legacy pathways (Weed 2009). The two potential legacy pathways for change have been reported to be, firstly, a ‘demonstration’ effect, and secondly a ‘festival’ effect (Weed 2009). Previous research in the field has stated that legacy pathways may influence behaviours at grassroots level, particularly among young children and/or adolescents (Ramchandani, Kokolakakis, and Coleman 2014). However, research in the field is lacking and the longevity of potential effects has yet to be established (Ramchandani, Kokolakakis, and Coleman 2014).

The ‘demonstration effect’ can operate in one of two ways: firstly, indirectly as a supporting mechanism, through the development and/or regeneration of sporting and/or community facilities; or, secondly, directly as when the achievements of athletes ‘trickle-down’ within a system (top-bottom), inspiring individuals to participate in sport and/or physical activity (Hindson, Gidlow, and Peebles 1994; Sotiriadou, Shilbury, and Quick 2008; Weed 2009; Wicker and Sotiriadou 2013). This ‘demonstration effect’ works on the assumption that a mega-sport event encourages individuals to: increase physical activity, instigate sport participation; motivate a change in frequency, intensity and/or duration of current sports participation; or increase their level of interest or change their attitude/s towards physical activity and/or sport (Weed 2009; Wicker and Sotiriadou 2013). This has been further explained through an adapted version of the trans-theoretical model (TTM), which is the most commonly used model that relates to participation in exercise and physical activity (Hillsdon, Foster, and Thorogood 2005; Mair and Laing 2013; Ramchandani et al. 2015). Ramchandani et al. (2015) noted that the first stages of the TTM (pre-contemplation, contemplation and preparation) are the ‘most susceptible to messages delivered through events’ and the stages that the ‘demonstration effect’ can have the greatest impact on; whilst the latter stages of the adapted model (action and maintenance) can be brought about by subsequent interventions implemented in the community alongside and/or following an event. The ‘demonstration effect’ is said to be most relevant to those people who are already physically active, or are currently or have, previously participated in sport, whereas more work is required in addition to the ‘demonstration effect’ of an event in order to assist those individuals who would be considered as sedentary to change their lifestyle behaviours (Ramchandani et al. 2015).

The second legacy pathway that has been identified in previous research is the ‘festival effect’ or ‘social leveraging’ of a mega-sport event (Chalip 2006; Weed 2009) which relates to a potential increase in the desire of individuals to be involved with and participate in a collective, enjoyable event such as the Commonwealth Games, Olympics or FIFA World Cup (Weed 2009). As with the ‘demonstration effect’, and in accord with the trans-theoretical model of behaviour change, the ‘festival effect’ is also deemed to be more relevant to the least physically active groups of the population, and will assist in producing a ‘nudge’ or shift towards contemplating participating in sport, becoming more physically activity or becoming physically active often for the first time (Prochaska and Velicer 1997).

### The Glasgow commonwealth games 2014

Previous research has suggested that mega-sporting events such as the Commonwealth Games, Olympics or FIFA World Cup could act as population-level interventions prompting physical activity, exercise and sporting behaviour change through the legacy pathways stated above, however the evidence is limited (Horne and Manzenreiter 2006; Preuss 2007; McCartney, Hanlon, and Bond 2010; Veal, Toohey, and Frawley 2012). A main driver behind Glasgow submitting a bid to host the 2014 Commonwealth Games was that the health and well-being of Glasgow’s residents is among the poorest in Europe, even after taking account of deprivation (Walsh et al. 2010). This phenomenon is termed the ‘Glasgow Effect’ (Walsh et al. 2010). It was felt that the potentially beneficial health improvements of an increase in sports participation and physical activity levels and a decrease in sedentary behaviour as legacy objectives which might accrue from Glasgow hosting a mega-sport event may be healthful for the city’s residents, instigated through the legacy pathways (Walsh et al. 2010; Scottish Government Social Research 2012).

In the lead up to Glasgow Commonwealth Games 2014, the Scottish Government developed a 10-year legacy plan detailing four domains—active, flourishing, sustainable and connected—with the potential for long-term legacy (Kemlo and Owe 2014). It was the aim of the Scottish Government that the Games would not just be a one off major sporting event hosted by the city but would provide Glasgow, and outwardly the whole of Scotland, with a lasting beneficial legacy in terms of the four stated domains (Scottish Government 2012). The first established domain ‘Flourishing’ was included to enable Scotland and more specifically Glasgow to benefit economically from the Games in both the short- and long-term. ‘Connected’ was included to establish domestic and international cultural links. ‘Sustainable’ was seen as an important domain through which to educate the host nation on environmental issues and promote sustainable living through demonstration projects such as the Athletes’ Village. The final component was the ‘Active’ legacy domain. This health and well-being domain was established to improve the lives of those who live in Scotland and to help them become more physically active (Scottish Government 2012).

Mega-sporting events are often supported by additional legacy programmes to help boost event impacts on physical activity and sports participation, in accord with the argument about the TTM and a ‘demonstration effect’ (Ramchandani et al. 2015). In the case of the Glasgow Commonwealth Games 2014, the primary legacy programme related to sports infrastructure, and consisted of £198 million being spent on new and refurbished sporting facilities, mostly in the East End of the city, including the refurbishment of Tollcross International Swimming Centre and the construction of the National Hockey Centre, the Emirates Indoor Arena and the Sir Chris Hoy Velodrome (Clark and Kearns 2016). Other club- and community-based legacy programmes were also instigated including training new coaches to work with local sports clubs, the development of Community Sports Hubs which aimed to not only increase but also to create opportunities for Scottish people to become active, and making Glasgow more accessible by improving the city’s transport and active travel infrastructure, including several new cycle lanes (Clark and Kearns 2016).

### Research aim

Our focus here is on the ‘Active’ legacy domain, which aimed to help the residents of Scotland become more physically active and lead healthier lifestyles (Scottish Government Communities Analytical Services (CASD) and the Games Legacy Evaluation Working Group 2014). Hence, the aim of the current study was to determine if, following a mega-sport event (Commonwealth Games Glasgow 2014), positive changes were reported regarding behaviour and attitudes towards sport and physical activity by individuals living in the nearby host community, through either of the two theorized mega-sport event legacy pathways, namely the demonstration effect and/or the festival effect.

## Methods

Data for the current study were collected as part of the longitudinal GoWell East: Studying Change in Glasgow’s East End study. This study is part of the longitudinal multi-disciplinary inter-sectoral GoWell Project (Egan et al. 2010; Cleland et al. 2015). The GoWell Project is a ten-year research and learning programme that aims to investigate the impact of investment in housing and neighbourhood regeneration on the health and well-being of those who live in Glasgow, Scotland (Egan et al. 2010; Cleland et al. 2015). More specifically the GoWell East study was designed in order to determine the impacts of the Glasgow Commonwealth Games 2014. In addition, the study was designed to investigate the impacts of regeneration that occurred as a result of the Games on the health and life chances, of residents, of the rapidly changing East End of Glasgow (Cleland et al. 2015). As the East End of Glasgow was home to the majority of the Commonwealth Games facilities and the newly constructed Athletes Village, the area has been subject to considerable infrastructure, amenity, social and economic change from 2007 up to and beyond the Games in 2014 (Cleland et al. 2015; Clark and Kearns 2016; Clark, Kearns, and Cleland 2016).

### Study area

**Figure 1.**
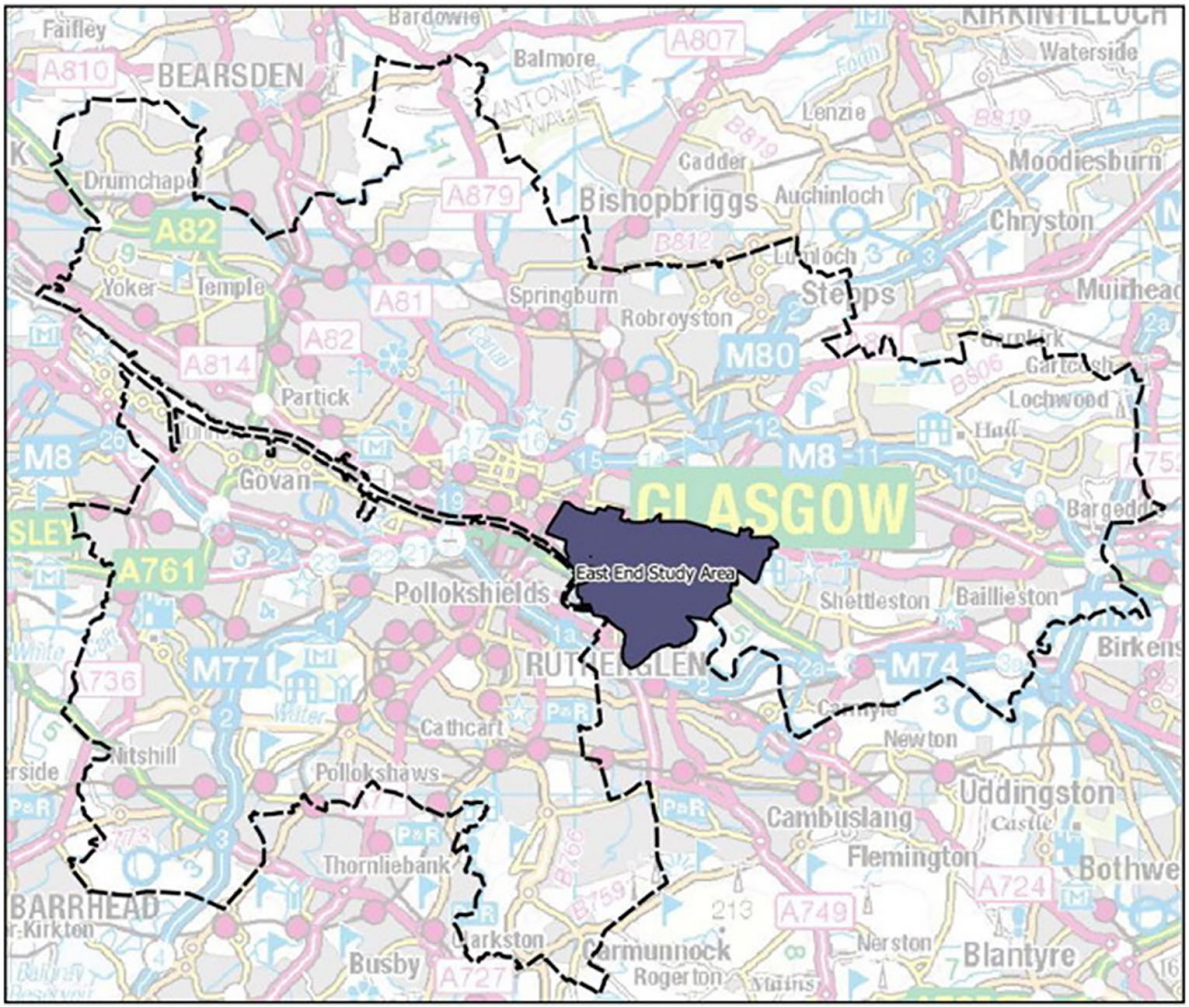
Glasgow district, outlining the East End Study Area (Clark et al. 2016).

The East End of Glasgow which was the site of the 2014 Commonwealth Games has a population of approximately 19,000 residing in approximately 10,000 homes across six local areas: Bridgeton, Calton, Camlachie, Dalmarnock, Gallowgate and Parkhead. The study area was located just east of the city centre (Figure 1) and is coterminous with the East End Development Strategy Area declared for regeneration purposes by Glasgow City Council (GCC 2008; GCC 2014).

The study area has a complex dynamic regarding housing tenure, with the level of social housing declining over the past two decades and the level of private renting increasing; indeed, by 2011 a quarter of households in the study area were privately renting, more than is the case in the city as a whole. It should also be noted that the neighbourhoods that comprise the study area are extremely ‘hard-to-reach’ in behaviour change terms as they are among the most deprived neighbourhoods in Scotland, lying within the 8th and 10th deciles of income deprivation in Scotland (Walsh 2008). Furthermore, there is a higher than average transient population due to the extent of rented housing (http://www.gowellonline.com/goeast) (Clark and Kearns 2013; Bonevski et al. 2014).

### Data source

The GoWell East Study is a longitudinal study with the first survey wave being implemented in May-August, 2012, approximately two years prior to the Commonwealth Games Glasgow 2014. The second survey wave was implemented in October-February, 2014, designed to collect responses as close to the end of the Commonwealth Games as possible (2–6 months’ post-event) and following the reopening of sporting and leisure facilities following the Games (Cleland et al. 2015).

During wave one of the GoWell East Study, 1015 adults were interviewed face-to-face within their homes in the East End of Glasgow by a trained field worker from the Medical Research Council Glasgow Survey team (Clark and Kearns 2013). In order to produce a longitudinal dataset each participant from GoWell East wave one was considered eligible for potential participation at wave two. Prior to re-contacting participants from wave one, data linkage was employed via each consenting participant’s community health index (CHI) number in a bid to obtain their most up to date address (Cleland et al. 2015). Following data linkage, postal invitations for wave two were sent in a phased approach by community (Bridgeton, Calton, Camlachie, Dalmarnock, Gallowgate and Parkhead) to each potential participant’s most up to date address or their last known address.

Following the delivery of postal invitations, trained field workers attempted to make contact with participants via email, telephone or by visiting the participant’s home (Cleland et al. 2015). Criteria for inclusion within the study at wave one were: 16 years of age or older; currently responsible for paying a mortgage, owning their own home, or renting their home as a social sector tenant or a private sector leaseholder; and the sole or joint householder or partner thereof, residing in the dwelling (Cleland et al. 2015). The second wave of the GoWell East Study comprised 414 previously interviewed participants from wave one, thus producing a longitudinal dataset (*n* = 414) with a response rate of 41%.

The GoWell East survey asked participants about their demographic characteristics, their household, the neighbourhood they reside in, their physical health, their mental health and wellbeing, their level of physical activity and sports participation, if they have any involvement with cultural activities, and their attitudes towards the Commonwealth Games Glasgow 2014.

### Study measures

#### Outcome measure

The main outcome measure for the current study was self-reported change in attitudes towards sport and/or physical activity or change in reported behaviour, at Wave 2. Within the GoWell East survey wave two participants were asked whether the Glasgow Commonwealth Games 2014 had influenced them in any of the following ways: (a) I have taken up a new sport; (b) I am thinking about taking up a new sport; (c) I am doing more sport or physical activity; (d) I am thinking about doing more sport or physical activity; (e) I am more interested in sport and physical activity in general; or (f) none.

Data preparation for the main outcome variable involved grouping participant responses to produce a hierarchical dependent variable for behaviour change with four levels: (3) Changed Behaviour: I have taken up a new sport (a) or, I am doing more sport or physical activity (c); (2) Contemplating Change: any individual who was not included in (3) but who gave one of the following responses: I am thinking about taking up a new sport (b); I am thinking about doing more sport or physical activity (d); (1) Pre-Contemplative: any individual who was not included in (3) or (2) but who gave the following response: I am more interested in sport and physical activity in general (e); or (0) No Change: any individual who was not included in (3), (2) or (1) but who gave the following response: none of the above (f).

#### Independent variable

Participants were asked during wave two to rank on a five point Likert scale ranging from ‘very important’ (1) to ‘very unimportant’ (5) their views on how important different elements of the mega-sport event were to them as an experience. This included: (a) the medal winning performance of Scottish and Home Nations athletes; (b) seeing world-class athletes from around the world compete; (c) the atmosphere and enjoyment of the sports events; (d) the atmosphere and enjoyment of the cultural events and entertainment around the city; and (e) the integration of Paralympic-sports within the main Games.

Three independent variables were included within the analysis performed as part of the current study and were derived from: (1) Demonstration Effect: participants who responded ‘very important’ to: items a (domestic achievement), b (international performance) or e (integration of Paralympic-sports); (2) Festival Effect: participants who responded ‘very important’ to: items c (atmosphere of sports events) or d (atmosphere of cultural events); or (3) Combined Demonstration and Festival Effects: participants who answered ‘very important’ for both the Demonstration and Festival Effects.

Reporting that the Demonstration, Festival or a combination of both effects were less than ‘very important’ was the reference category. The top response categories of ‘very important’ were selected as the predictive items of interest; in what follows we refer to the relevant effect being ‘important’ for ease of reference. The rationale for doing this was in the light of the fact that before the Commonwealth Games, there was extensive popular support for and interest in the Games in Glasgow. Approximately 75% of adults were reported to be supportive of the mega-sport event that was due to take place in their city (Scottish Government Communities Analytical Services (CASD) and the Games Legacy Evaluation Working Group 2014). Thus, it was likely that most respondents in wave two of the GoWell East Study would respond that aspects of the Games were important to them, and therefore we sought to identify those people for whom the identification with the mega-sport event was strongest and above average.

#### Socio-demographic characteristics

Additional variables were collected during wave two and were used in the analysis as known correlates for physical activity. Such variables of interest were: (1) age group in four groups (≤35; 36–50; 51–64; 65+); (2) gender; (3) employment status (full-time, part-time, full-time education, long-term sick/disabled, not working [unemployed; temporary sick; looking after home; other], retired); and (4) education (college-level/equivalent, school-level/equivalent, none).

#### Baseline variables

In order to isolate the effect of the ‘intervention’ of a mega-sport event, baseline sports participation and level of physical activity prior to the Games taking place in Glasgow were included within the statistical analysis. Participation in sport over the past four weeks was reported by participants at Wave 1 by responding ‘yes/no’ to a list of forty different sports (Table 1). From this, a binary variable of baseline participation in sport (or not) was constructed. Level of physical activity at baseline was measured during wave one by the international physical activity questionnaire (IPAQ) (Craig et al. 2003; Lee et al. 2011). As the IPAQ provides minutes of moderate-to-vigorous physical activity, participants were classified as having low (–0.5 Standard Deviation), moderate (mean) or high (+0.5 Standard Deviation) physical activity (IPAQ 2014; Patterson 2010).

**Table 1.** List of sporting activities (Wave 1).

Aerobics/keep fit/gymnastics/dance (for fitness)Badminton/tennisCyclingDancing (other types)Exercises (e.g. press-ups, sit-ups)Football/rugbyGym (workout)/exercise bike/weight training running/joggingSquashSwimming AthleticsAquarobics/aquafit/exercise class in waterBasketballBowls	BoxingCanoeing/kayakingClimbingCricketCurling Fishing/anglingGolfHill walking/ramblingHockeyHorse ridingIce skatingMartial arts (including Tai Chi)NetballPowerboating/jet skiing	RowingSailing/windsurfing Shinty/gaelic football Skateboarding/inline skating Snooker/billiards/poolSkiing/snowboardingSubaquaSurfing/body boardingTable tennisTenpin bowlingVolleyballWaterskiingYoga/pilatesOther

### Statistical analysis

Basic descriptive analysis was performed to establish the distribution of the outcome measure, independent variables, demographic characteristics and baseline variables. Frequency distributions were then examined by gender for the two baseline variables (sports participation and level of physical activity). The outcome measure and independent variables at wave two were then cross-tabulated to examine relationships within the data.

Spearman’s Rank correlations were then performed to determine which of the independent variables and if any of the baseline variables had a significant relationship with the outcome measure.

Multinomial logistic regression models were then constructed for the outcome variables of pre-contemplative, contemplative and behaviour change with no change set as the reference. The models only included variables that were found to have a statistically significant relationship with the outcome measure following Spearman’s Rank correlations. Data were inserted into the models to ensure that the reference category for each independent variable was the ‘less than very important’ category and for the socio-demographic and baseline variables the reference category was set at the least effective level within the variable regarding physical activity and sports participation according to previous research, these being: female; aged 65+; retired; no qualifications; no sports participation; and low level of physical activity.

### Ethical approval

Ethical approval was provided by the NHS Scotland A REC committee (no. 05/MRE10/89). At both time-points of the study GoWell East participants provided written informed consent.

## Results

Results showed the sample was predominately female (61.1%), aged 30–64 years (59.1%), had school/college qualifications (73.6%) and was mostly comprised of the employed (full/part-time) (39.1%) and retired (33.3%) (Table 2). At baseline, a third of respondents (33.1%) had a low level of physical activity, half (51.0%) had a moderate level and nearly one-in-seven (15.5%) had a high level (Table 3). Levels of physical activity were similar between men and women. In addition, at baseline over half the respondents, 55.8%, participated in sport, close to the national average of 54% (Scottish Government 2012) (Table 3).

**Table 2.** Demographic characteristics of the longitudinal cohort at Wave 2.

	Percentage (%)	*Frequency (n)*
*Gender*		
Male	38.9	161
Female	61.1	253
*Age*		
16–29	19.1	22
30–49	30.3	118
50–64	28.8	149
65+	18.6	122
Unrecorded	3.1	3
Educational attainment*		
College level qualifications	41.5	172
School qualifications or equivalent	32.1	133
None	25.6	106
Missing	0.8	3
*Employment status*		
Full time work	26.8	111
Part time work	12.3	51
Training	0.0	0
Full time education	2.7	11
Unemployed	5.6	23
Temporarily sick	1.7	7
Long-term sick/disabled	12.8	53
Looking after home	3.4	14
Other	1.2	5
Retired	33.3	138
Not recorded	0.2	1

*College level (B Tech or diploma; advanced diploma; HNC or HND; first or higher degree), school qualifications or equivalent (school leaving cert; GCSE d–f; GCSE a–c; A levels; apprenticeship or trade; other technical/business; other qualification); or none.

**Table 3.** Level of physical activity and sport participation at Wave 1.

	Entire Sample		Males		Females	
	%	*n*	%	*n*	%	*n*
Low level of physical activity	33.1	137	37.9	61	30.0	76
Moderate level of physical activity	51.0	211	46.0	74	54.2	137
High level of physical activity	15.5	64	16.1	26	15.0	38
Missing	0.5	2			0.8	2
Participated in sport	55.8	231	60.2	97	53.0	134

The theorized legacy pathways were found to be relevant for a large proportion of the sample. As Table 4 shows, the Demonstration Effect was ‘important’ for nearly two-thirds of respondents (63.3%) the Festival Effect was ‘important’ for three-in-five (59.2%) and the Combined Effect was ‘important’ for half the respondents (50.7%). In terms of behaviour change following the Commonwealth Games, a small number of people, 7.5%, reported that they had changed their sport/physical activity behaviour, one-in-ten respondents (10.1%) were contemplating changing their behaviour and a further one-in-ten (10.6%) were classified as pre-contemplative; the majority of respondents (71.7%) reported no change (Table 5).

**Table 4.** Distribution of the independent variables.

Variable	Frequency (*n*)	Percentage (%)
*Demonstration effect*		
Not important	152	36.7
Important	262	63.3
Total	414	100.0
*Festival effect*		
Not important	169	40.8
Important	245	59.2
Total	414	100.0
*Combined effect*		
Not important	204	49.3
Important	210	50.7
Total	414	100.0

**Table 5.** Distribution of self-reported change in sport and/or physical activity behaviour or change in attitude towards sport and/or physical activity.

	No change n (%)	Pre-contemplating Change n (%)	Contemplating Change n (%)	Change Behaviour n (%)
Overall sample	297 (71.7)	44 (10.6)	42 (10.1)	31 (7.5)
*Gender*				
Male	113 (70.2)	18 (11.2)	14 (8.7)	16 (9.9)
Female	184 (72.7)	26 (10.3)	28 (11.1)	15 (5.9)
*Age*				
Up to 35	44 (56.4)	14 (17.9)	8 (10.3)	12 (15.4)
36–50	53 (58.2)	11 (12.1)	16 (17.6)	11 (12.1)
51–64	111 (78.7)	10 (7.1)	13 (9.2)	7 (5.0)
65+	87 (86.1)	9 (8.9)	4 (4.0)	1 (1.0)
Unrecorded	2 (66.7)	0 (0)	1 (33.3)	0 (0)
Educational attainment*				
College level qualifications	101 (58.7)	28 (16.3)	21 (12.2)	22 (12.8)
School qualifications or equivalent	99 (74.4)	10 (7.5)	18 (13.5)	6 (4.5)
None	94 (88.7)	6 (5.7)	3 (2.8)	3 (2.8)
Missing	3 (100.0)	0 (0)	0 (0)	0 (0)
*Employment status*				
Full/part time work/training/education	98 (56.6)	26 (15.0)	26 (15.0)	23 (13.3)
Not working	34 (69.4)	6 (12.2)	8 (16.3)	1 (2.0)
Long-term sick/disabled	43 (81.1)	2 (3.8)	1 (1.9)	7 (13.2)
Retired	121 (87.7)	10 (7.2)	7 (5.1)	0 (0)
Not recorded	1 (100.0)	0 (0)	0 (0)	0 (0)

*College level (B Tech or diploma; advanced diploma; HNC or HND; first or higher degree), school qualifications or equivalent (school leaving cert; GCSE d–f; GCSE a–c; A levels; apprenticeship or trade; other technical/business; other qualification); or none.

When the outcome measure was assessed by demographic characteristics, the results showed that a higher percentage of males changed their behaviour (9.9%) and pre-contemplated changing their behaviour (11.2%), although in terms of contemplating behaviour change a higher percentage of females was found (11.1%) (Table 5). Results by age, educational attainment and employment status showed trends as expected: those who were younger (under 35 years), had a college level education or equivalent, and were employed, in education or training had changed their behaviour or pre-contemplated changing their behaviour more so than other people. Conversely, participants aged 65 or more, or who were retired or who had no educational qualifications, were the least likely to report any kind of change in behaviour (Table 5).

Cross-tabulations revealed modest levels of potential interaction between behavioural or attitudinal change and the experience of the mega-sport event itself. Of those who deemed the demonstration effect important to them, only 8.0% reported a change in behaviour. Similarly, of those who felt that the festival effect was important to them, only 9.4% had changed their behaviour. Lastly, 9.0% of those who felt the combined demonstration and festival effect was important to them reported a change in behaviour (Table 6). However, around a quarter (25–29%) of those who identified any of the three legacy pathways as important to them reported pre-contemplative or contemplative changes in behaviour (Table 6). Thus, the majority (between three fifths and four fifths) of those who deemed the legacy pathways important to them, did not report any changes in sport or physical activity attitudes or behaviours.

**Table 6.** Cross tabulations of the study outcome measure and independent variables.

	No change *Level 0*	Pre-contemplating change *Level 1*	Contemplating change *Level 2*	Changed behaviour *Level 3*	Total
*Demonstration effect*					
Not important	122	12	8	10	152
% within demonstration effect	80.3%	7.9%	5.3%	6.6%	100.0%
% within outcome measure	41.1%	27.3%	19.0%	32.3%	36.7%
Important	175	32	34	21	262
% within demonstration effect	66.8%	12.2%	13.0%	8.0%	100.0%
% within outcome measure	58.9%	72.7%	81.0%	67.7%	63.3%
*Festival effect*					
Not important	144	7	10	8	169
% within demonstration effect	85.2%	4.1%	5.9%	4.7%	100.0%
% within outcome measure	48.5%	15.9%	23.8%	25.8%	40.8%
Important	153	37	32	23	245
% within demonstration effect	62.4%	15.1%	13.1%	9.4%	100.0%
% within outcome measure	51.5%	84.1%	76.2%	74.2%	59.2%
*Combined effect*					
Not important	166	15	11	12	204
% within demonstration effect	81.4%	7.4%	5.4%	5.9%	100.0%
% within outcome measure	55.9%	34.1%	26.2%	38.7%	49.3%
Important	131	29	31	19	210
% within demonstration effect	62.4%	13.8%	14.8%	9.0%	100.0%
% within outcome measure	44.1%	65.9%	73.8%	61.3%	50.7%

Spearman’s Rank correlations showed that of the socio-demographic and baseline variables, age, employment status, highest educational attainment, sports participation and physical activity level at baseline all had significant relationships with behaviour and attitudinal change, whilst gender had a non-significant relationship (*p* > 0.05) (Table 7). All three-legacy pathway variables held significant bivariate associations with the outcome measure of attitudinal and behaviour change.

**Table 7.** Spearman’s rank correlations between attitudinal and behavioural change (outcome measure) and independent, socio-demographic and baseline variables.

Variable	Correlation coefficient	Level of significance
Age	–0.274	0.000
Gender	–0.033	0.497
Employment Status	0.304	0.000
Highest Educational attainment	0.268	0.000
Sports participation at baseline	0.190	0.000
Physical activity level at baseline	0.185	0.000
Demonstration effect	0.140	0.004
Festival effect	0.236	0.000
Combined effect	0.205	0.000

On the basis of the previous analysis, a multinomial regression model was developed to examine whether the selected independent variables were associated with each level of attitudinal and behaviour change (Table 8). Of the legacy pathway variables, no effects were found for either the demonstration effect or the combined demonstration and festival effect (Table 8). However, a significant result was found for the festival effect of a mega-sport event and one of the outcome measures of behaviour and attitudinal change: respondents were nearly seven times more likely to be in the pre-contemplative change group than the no change group if they reported that the festival effect of the mega-sport event was important for them (*p* < 0.006).

**Table 8.** Multinomial logistic regression.

		Std. error	Sig.	Exp(B)	95% Confidence interval for Exp(B)	
					Lower bound	Upper bound
Level 1 Pre-contemplative	Intercept	1.025	0.000			
	Demonstration effect (important)	0.812	0.322	20.233	0.455	10.964
	Demonstration effect (not important)					
	Festival effect (important)	0.688	0.006	6.595	1.711	25.420
	Festival effect (not important)					
	Combination effect (important)	0.944	0.343	0.409	0.064	2.601
	Combination effect (not important)					
	Age (65+ years)	0.822	0.862	0.867	0.173	4.341
	Age (51–64 years)	0.522	0.116	0.441	0.159	1.225
	Age (36–50 years)	0.488	0.669	0.812	0.312	2.112
	Age (up to 35 years)					
	Employment (working/training/education)	0.841	0.148	3.372	0.648	17.532
	Employment (not working)	0.919	0.398	2.174	.359	13.163
	Employment (retired)	0.978	0.872	1.171	.172	7.956
	Employment (long-term sick/disabled)					
	Education (college level or equivalent)	0.542	0.047	2.937	1.014	8.505
	Education (school level or equivalent)	0.575	0.902	1.073	0.347	3.315
	Education (none)					
	Sports participation baseline (yes)	0.418	0.079	0.479	0.211	1.089
	Sports participation baseline (no)					
	Physical activity level (high)	0.586	0.964	0.974	0.309	3.069
	Physical activity level (moderate)	0.428	0.723	1.164	0.503	2.694
	Physical activity level (low)					
Level 2Contemplative	Intercept	1.301	0.000			
	Demonstration effect (important)	0.744	0.699	1.334	0.311	5.729
	Demonstration effect (not important)					
	Festival effect (important)	1.108	0.383	0.380	0.043	3.339
	Festival effect (not important)					
	Combination effect (important)	1.287	0.163	6.025	0.483	75.143
	Combination effect (not important)					
	Age (65+ years)	0.902	0.654	.668	0.114	3.910
	Age (51–64 years)	0.543	0.832	1.122	0.387	3.251
	Age (36–50 years)	0.507	0.130	2.155	0.797	5.828
	Age (up to 35 years)					
	Employment (working/training/education)	1.089	.099	6.021	.713	50.850
	Employment (not working)	1.134	0.105	6.285	0.681	57.997
	Employment (retired)	1.190	0.337	3.137	0.304	32.325
	Employment (long-term sick/disabled)					
	Education (college level or equivalent)	0.677	0.042	3.968	1.053	14.953
	Education (school level or equivalent)	0.682	0.041	4.023	1.058	15.303
	Education (none)					
	Sports participation baseline (yes)	0.428	0.903	0.949	0.410	2.197
	Sports participation baseline (no)					
	Physical activity level (high)	0.633	0.462	10.593	0.461	5.502
	Physical activity level (moderate)	0.494	0.195	10.897	0.720	4.998
	Physical activity level (low)					
Level 3Behaviour change	Intercept	1.060	0.001			
	Demonstration effect (important)	0.913	0.720	1.386	0.232	8.294
	Demonstration effect (not important)					
	Festival effect (important)	0.760	0.569	1.541	0.347	6.839
	Festival effect (not important)					
	Combination effect (important)	1.130	0.766	1.399	0.153	12.809
	Combination effect (not important)					
	Age (65+ years)	1.350	0.817	1.368	.097	19.264
	Age (51–64 years)	0.563	0.134	0.430	0.143	1.297
	Age (36–50 years)	0.514	0.977	1.015	0.371	2.779
	Age (up to 35 years)					0
	Employment (working/training/education)	0.656	0.040	0.260	0.072	0.939
	Employment (not working)	1.189	0.005	0.036	0.003	0.367
	Employment (retired)	0.000		1.722E–10	1.722E–10	1.722E–10
	Employment (long-term sick/disabled)					
	Education (college level or equivalent)	0.807	0.146	3.231	0.665	15.699
	Education (school level or equivalent)	0.839	0.974	0.973	0.188	5.038
	Education (none)					
	Sports participation baseline (yes)	0.672	0.182	2.453	0.657	9.159
	Sports participation baseline (no)					
	Physical activity level (high)	0.848	0.083	4.359	0.827	22.991
	Physical activity level (moderate)	0.760	0.037	4.896	1.105	21.703
	Physical activity level (low)					

The regression model showed no significant effects upon the outcome measure for age or for baseline sport participation. However, education level was found to be important for some outcomes. College education or equivalent was significantly associated with both pre-contemplative and contemplative change (*p* < 0.05) but not with behaviour change. School level education or equivalent was also found to be significantly associated with an increase in the likelihood of contemplative change (*p* < 0.05). Employment status was significantly associated with reported behaviour change. Although similar proportions of those in work and long-term sick or disabled reported behaviour change in the survey (13% each—see Table 5), when other factors were taken into account, those in employment were less likely to report behaviour change than those who were long-term sick or disabled (*p* < 0.05), although all three groups—those working, not working and long-term sick or disabled—were more likely to report behaviour change than those retired (Table 8). Finally, those who reported a moderate level of physical activity at baseline were significantly more likely—by nearly five times—to report behaviour change than those who had a low level of prior physical activity (Table 8).

## Discussion

We set out to examine the operation of the hypothesized ‘demonstration’ and ‘festival’ effects from a mega sport event upon behaviour and attitudes towards sport and physical activity by individuals living in the nearby host community. The study strengths include the analysis of a longitudinal data-set that included specific questions aimed at identifying which aspects of the event were important for individuals residing in the community closest to the mega sporting event site, enabling the identification of the two theorized mechanisms. This, as far as we know, is the first time such an approach has been taken to study the effects of a mega sport event; evidence for the proposed mechanisms and the health effects of mega-sport event are generally sparse or absent (McCartney, Hanlon, and Bond 2010, 2013).

Moreover, we have studied the community residing in the core hosting area for a mega sport event, namely the Commonwealth Games 2014 in Glasgow. This is a deprived community with traditionally low levels of sport participation and physical activity: prior to the Games, approximately 40% of adults had low physical activity levels (<30 min moderate-to-vigorous physical activity per week) (Clark and Kearns 2013). As such, this is the area in which policy-makers would wish to have an impact upon health behaviours (Leadbetter, Geyer, and O’Connor 2014). The place in question is also the area provided with the newest opportunities for sports participation and physical activity through the provision of new/improved facilities and infrastructure for the mega sport event, thus providing the supporting infrastructure for the hypothesized mechanisms to take effect, if and when, they are operative (Clark and Kearns 2015).

Investigating reported changes in behaviour and attitudes toward sport and physical activity by individuals living in the nearby host community following the mega-sport event, or the contemplation of such change, we found, firstly, that the demonstration and festival effects mechanisms were relevant to a large number of people, with around 60% rating the relevant aspects of the Games as important for them. Thus, we would say that the mechanisms were found to be operative.

However, the picture is less positive when we consider the effectiveness of the mechanisms, as a significant association with attitudinal change was only found for the Festival Effect. Respondents were more likely to be in the pre-contemplative change group (i.e. to be more interested in sport and physical activity) than no change if they reported that the festival effect of the mega-sport event (i.e. the atmosphere they experienced around the event) was important to them. No significant associations with attitudinal or behaviour change were found for either the Demonstration Effect or a combination of the Festival and Demonstration effects. Therefore, for most people for whom the mechanisms are relevant, they are not effective. This finding is in line with Weed et al. (2015) who reported that the demonstration effect of a mega sporting event fails to change earlier attitudinal stages of physical activity. Furthermore, results from the current study reinforce work by Ramchandani and colleagues who reference the conceptual models of participation and the link with mega events (Ramchandani et al. 2015); when the adapted TTM is considered within the context of the current study, the current results support the contention that mega events have the potential to positively impact the first three stages of change: (1) Pre-contemplation, (2) Contemplation and (3) Preparation (Mair and Laing 2013; Ramchandani et al. 2015).

When more in depth analysis was performed results from the current study indicated the importance of three other factors. Those with college-level education exhibited increased likelihood of post-Games attitudinal change (but not behaviour change) and by more than the Games-related mechanisms. In addition, school level education or equivalent was associated with an increase in the likelihood of contemplative change. The second factor associated with change post mega event was employment status; those individuals who were employed at the time of the mega-sport event were less likely to report behaviour change than those who reported to be long-term sick or disabled. In addition, analysis showed that those who were employed, unemployed or long-term sick or disabled, were more likely to report behaviour change than those who were retired. The third factor that was found to have a significant impact was physical activity level at baseline. Results showed that those individuals who reported a moderate level of physical activity at baseline were significantly more likely to report behaviour change post the mega sporting event than those who had a low level of physical activity before the mega sporting event.

These factors reinforce the argument that increasing physical activity and sports participation in a deprived population may depend upon more fundamental interventions to raise the prospects of positive change for a minority of the target group. This finding concurs with previously published research that reported that in order to bring about behaviour change as opposed to solely attitudinal change, additional strategies and programmes were required in addition to the mega sport event (Mair and Laing 2013; Ramchandani et al. 2015). The event alone would be insufficient to bring about both attitudinal and behavioural change. In this respect, it may be more beneficial and may prove more advantageous than the mega sport event itself in the medium-to-long term if sport and physical activity interventions were to be set up alongside the mega sport event. Furthermore, ‘cross-pathway’ activities, such as the delivery of socially based, physical activity and sport interventions through community venues and sports facilities may also offer good prospects of bringing about change (Clark and Kearns 2015).

This raises the question of how much the inspirational approach of the demonstration and festival effects matters to individuals pre-, during and post-mega sporting events versus or in combination with other means of encouraging physical activity and sports participation. The Glasgow Commonwealth Games attempted to encourage physical activity and sports participation in parallel with the mega sporting event by improving the city’s/country’s sporting infrastructure, implementing club and community programmes, training new coaches and making the city accessible by improving cycle lanes (Clark and Kearns 2014). This research shows that inspirational effects cannot solely be relied upon. Moreover, mega event stakeholders should be aware of ‘initiativitis’ and aim to leverage legacy through a balanced strategic approach of inspiration and practical proactive community/club programmes (Weed et al. 2015; Lovett and Bloyce 2017).

It is also evident from the current research that the promotional and public engagement strategy that was put in place for the Glasgow Commonwealth Games was extremely effective, with a high proportion of the population engaging and showing support for the games and reporting the importance of the festival, demonstration and combined effects. This finding highlights the need for future mega-sport events to replicate the engagement strategy adopted at the Glasgow Commonwealth Games. However, in addition, it would be recommended, that future mega-sport events further develop this strategy in order to ensure that they are not only gaining the support and engagement of the population who reside in a close proximity to the site of the mega-sport event; but they also effectively impact attitudes to, and sport and/or physical activity behaviour through the mega-sport event in the longer term.

An issue is working out how to extend the linked effect we have found—from enjoyment of the sports event itself (festival effect), through becoming more interested in sport (pre-contemplative change)—through to modest changes in physical activity and sports behaviours, particularly for those with low levels of activity. The fact that there are new sports facilities available in the locality offers an opportunity to build on the mega sport event in this way. However, in Glasgow’s case, there has been no major initiative to capture local resident interest and to promote free or heavily subsidised use of the new facilities. Indeed, research with local residents has revealed a reluctance to use the new facilities due to a mixture of cost, lack of appropriate facilities for the non-sporty, and a perceived unwelcoming atmosphere (Kidd, Clark, and Kearns 2017). Given the relevance of a festival effect for many adults, which we have shown, an alternative might be to initiate recruitment drives to increase participation both in sport and in leisure-based physical activities at future sports events held at the new venues, though again we are not aware of this happening.

It is also important to take into consideration the weighting of legacy objectives; and it should be noted that depending on the stakeholders involved with the mega-event planning committee, this may impact the weight each legacy objective is given and if they are weighted equally (Preuss 2007). Not only will the weighing of the legacy objectives influence the impact of each objective but so too will the conflicting interests of the stakeholders (Preuss 2007). The current mega sporting event under review had four legacy domains: ‘Flourishing’; ‘Connected’; ‘Sustainable’; and ‘Active’ (Scottish Government 2012). It might have been assumed that due to dwindling levels of physical activity, widening health inequalities and the ‘Glasgow Effect’ on health, it would have been the ‘Active’ legacy domain that received the highest priority within the Glasgow Commonwealth Games legacy effort. However, looking at results from the current research and a previous report on the Glasgow Commonwealth Games that showed there was no significant increase in physical activity or sports participation post games (Cleland et al. 2015) this may not have been the case.

When investigating the legacy of mega sporting events there is always the potential limitation of the effect of time. When it comes to legacy it may be too soon to see a beneficial impact on health or indeed conversely, it may be too late and research should be performed in the moment during a mega sport event in order to identify effects (Weed et al. 2015). Uncertainty about the temporal rhythms of legacy arises not only from what is known and currently presented in previously published research regarding behaviour change (which takes time) but also and from what Rogerson (2016) observes as a move towards front-loading and pre-timing legacy impacts, which then risks those legacies not being sustainable afterwards. More legacy research is therefore required to unpick the legacies and legacy timings of mega-sport events.

Moreover, it is also important to note the relatively small sample size and the locality of the current sample; which was limited to the residential area surrounding the Commonwealth Games Site in Glasgow. Furthermore, physical activity within the current study was measured subjectively; which is subject to inaccuracies due to participant recall and social desirability bias (Adams et al. 2005). Finally, sedentary behaviour was not an outcome of the current study, which could be considered a limitation, as mega sporting events may have the potential to not only influence physical activity but also sedentary behaviour.

## Conclusions

It cannot be denied that mega-sport events are inspirational and bring the host city and nation together as one. However, when it comes to evaluating mega sport events for the purpose of influencing attitudes and behaviours towards improving levels of physical activity and/or increasing numbers of those who participate in sport, mega-sport events do not seem to have the power to inspire and cause long-term positive changes.

The current research shows that by following a strategic promotional campaign similar to the one implemented for the Glasgow Commonwealth Games the mega-sport event can act as a catalyst to inspire engagement with the event but in order to inspire attitudinal and behavioural change during and following the mega-sport event there is a need for a balanced proactive and practical approach which runs in parallel and extends before, during and after the event to offer individuals opportunities for physical activity and sport participation that are relevant to them and their socio-economic status.

## Recommendations for future research, policy and practice

Our results suggest that there are a range of recommendations for future research, policy and practice. Our study was based on residents of the area surrounding the site of the Games and future research studies evaluating mega-sporting events could endeavour to recruit a larger, more wide spread sample in order to determine the impact not only within site surrounding the mega-sporting event but also further afield, including the wider city and beyond. Moreover, objective measures of physical activity and consideration of objective sedentary behaviour are warranted. However, objective measurement of physical activity and/or sedentary behaviour is challenging and costly to collect from large studies.

Through the current study a recommendation for future mega-events would be the implementation of complementary physical activity and sports interventions alongside the main sporting event (Cleland et al. 2012; Cleland et al. 2014). It is thought that this would have the potential for positive impact on the community. It would be plausible to recommend that alongside the implementation of a mega-sport event, multi-disciplinary teams design, develop and implement ‘cross-pathway’ activities within community centres or local sport facilities with the aim of positively impacting levels of physical activity, sport participation and ultimately health and well-being (McCartney, Hanlon, and Bond 2013). Findings from the current research study suggest that such activities may prove to have a more healthful impact than the delivery of a mega-sport event in the medium-to-long term (Clark and Kearns 2015). Finally, it is recognized that the current mega-sport event under review took place in Scotland, a developed country; and many events in the future may be hosted by developing/emerging countries. However, the findings from the current study suggest that the recommendations made from this study are transferable to both developed and developing countries and should focus their legacy aim/s on making physical activity and sport participation accessible to all, alongside the main mega-sport event regardless of individual or area socio-economic status.
